# The Water Load Test As a Measure of Gastric Interoception: Development of a Two-Stage Protocol and Application to a Healthy Female Population

**DOI:** 10.1371/journal.pone.0163574

**Published:** 2016-09-22

**Authors:** Zoé van Dyck, Claus Vögele, Jens Blechert, Annika P. C. Lutz, André Schulz, Beate M. Herbert

**Affiliations:** 1 Institute for Health and Behavior, Research Unit INSIDE, University of Luxembourg, Esch-sur-Alzette, Luxembourg; 2 Centre for Cognitive Neuroscience and Department of Psychology, University of Salzburg, Salzburg, Austria; 3 Department of Clinical Psychology and Psychotherapy, Eberhard-Karls-University, Tuebingen, Germany; ITALY

## Abstract

The sensitivity for one’s own internal body signals (i.e., interoception) has been demonstrated to play an important role in the pathogenesis of eating and weight disorders. Most previous measures assessing interoceptive processing have not, or only partly, captured perception of hunger and satiety cues, which is a core aspect of interoceptive deficits in eating disorders. In addition, methods used to measure sensitivity to gastric signals are heterogeneous and findings inconsistent. The primary aim of the present study was to establish a standardised test to measure gastric interoception, and to provide normative data using a non-clinical adult sample. The two-step Water Load Test (WLT-II) involves ingestion of non-caloric water until perceived satiation (step 1) and until maximum fullness (step 2). The WLT-II consists of several variables: Besides volumes of water ingested until satiation and maximum fullness expressed in ml, percentage of satiation to maximum fullness is calculated as an individual index of gastric interoception that is not confounded with stomach capacity. Ninety-nine healthy women participated in the study. Measures included the WLT-II, the heartbeat tracking test, a self-report questionnaire assessing subjective sensations, and the Eating Disorder Inventory-2. Twenty-eight participants underwent test-retest of the WLT-II. Results suggest that the WLT-II is a valid and reliable measure of gastric interoception. Importantly, satiation volume and percentage of satiation to maximum fullness were strongly positively related to self-reported bulimic symptoms, indicating that the WLT-II could emerge as a useful clinical tool to measure interoceptive processing in the field of eating disorders.

## Introduction

The sensitivity for one’s own internal body signals (i.e., interoception) has been demonstrated to be important for a broad range of cognitive and affective functions [1]. In the literature, interoception is commonly referred to as the psychosomatic connection between the body and the brain, conveying signals regarding the state of the internal body and its visceral organs [2]. Several lines of investigation have suggested that interoception plays an important role in the pathogenesis of eating and weight disorders, in particular with respect to deficits in the perception of hunger and satiety [3,4]. These interoceptive deficits have typically been investigated in relation to, or by proxy of, awareness of emotional states. A widely used measure in the field of eating disorders is the Interoceptive Awareness subscale of the Eating Disorder Inventory (EDI), which measures the self-reported ability to accurately detect and respond to emotional states [5]. Objective measures of interoception have typically focussed on people’s ability to perceive their own heartbeats [6–9]. These heartbeat perception tasks have been demonstrated to represent well validated measures, as evidenced by relationships between cardiac perception accuracy and activation in brain structures responsible for the mapping of internal bodily responses, in particular the right anterior insula, and the somatomotor and cingulate cortices [9].

In the field of eating disorders, however, studies using heartbeat detection tasks have often yielded inconsistent results. Some studies reported attenuated interoceptive cardiac accuracy in anorexia nervosa [10] and in bulimia nervosa even after recovery [11], while others found no difference between eating disordered individuals and healthy controls [12]. One study even reported an enhanced cortical representation of afferent signals from the cardiovascular system, as indicated by increased heartbeat evoked potentials in the electroencephalogram of participants with anorexia nervosa [13]. These inconsistencies might be explained by the increased levels of anxiety and/or depression in eating disordered patients, as evidenced by high rates of comorbid mood and anxiety disorders [14,15], which are themselves often associated with altered cardioceptive accuracy [16–18]. In addition, a core aspect of interoceptive deficits in eating disorders, i.e., a specifically impaired perception of hunger and satiety cues, is not directly captured by measuring cardioceptive accuracy. Hence, to advance our understanding of interoception in the eating domain, more specific interoception measures focusing on the gastric tract are required.

Interoceptive processes in the gastric system can be assessed using different methods of distention of the stomach, most of which have been primarily used in patients with functional gastrointestinal disorders [2]. Gastric distention activates vagal afferents, which send signals from the stomach to the brain and lead to the perception of satiety and fullness, thereby regulating food intake [19]. Reduced sensitivity to gastric signals could result in disordered eating behaviours, such as excessive food intake [20]. Previous studies using gastric distention methods found larger gastric volume capacities in bulimic compared to healthy subjects [21,22], combined with reduced sensitivity to gastric distention [23]. These results are, however, based on invasive measures (e.g., barostat with intragastric balloon), which are cumbersome and unpleasant techniques that lack ecological validity [24–26].

Non-invasive and more participant-friendly methods are water load tests (WLTs), which have been originally developed to induce gastric distention and to assess gastrointestinal symptoms in patients with functional digestive disorders. WLTs stimulate the stomach using a natural distention stimulus (i.e., ingestion of water) and without the complex hormonal response of a caloric meal [25,27]. Initial studies have shown that WLTs are acceptable to both healthy individuals and patients with gastrointestinal symptoms, such as nausea, early satiety, and bloating, that are frequently experienced by bulimic patients [28]. Furthermore, WLTs have been demonstrated to be reproducible [27], are related to invasive, barostat measures [29,30], and the volume of water ingested represents a valid indicator of feelings of subjective fullness [26,27]. Importantly, cross-modal interoceptive convergence has been reported: the amount of water consumed during an adapted WLT protocol has been demonstrated to be negatively related to individual cardiac accuracy in healthy, normal-weight women [24]. These findings indicate that interoceptive sensitivity for cardiac and gastric signals overlaps in healthy persons, suggesting that there exists a generalized tendency to be aware of visceral events.

Although these findings suggest that WLT protocols represent a promising and valid way to assess gastric signal perception, they were initially developed to investigate gastric sensation and accommodation in patients with functional digestive disorders and would, therefore, benefit from some adaptations to measure eating-related gastric processing. An important limitation of current use of WLTs concerns the lack of standardisation in procedures and instructions [25]. For example, terms used to instruct the drinking procedure and the cessation of water ingestion have been used quite heterogeneously. The standardized definition of drinking thresholds, however, is of major importance to ensure comparability of results from different studies. We will use the term satiation, which is defined as “the process that leads to the termination of eating and that is accompanied by a feeling of satisfaction” [31]. This instruction was chosen in order to put a focus on eating-related gastric sensations that are both accessible and reproducible, but without the caloric intake of a meal. Furthermore, this instruction implies that, at least in healthy individuals, satiation is associated with a positive sensation of satisfaction, in contrast to the fullness that may be induced by gastric distention and has been assessed in most previous studies. Eating disordered or obese individuals, in contrast, tend to override satiation signals; they do not stop eating when they are comfortably full and continue to eat until feeling overstuffed [32]. Hence, from a clinical point of view, detection of satiation signals might be a very relevant information going beyond the stomach fullness as assessed in initial WLT protocols.

Another important limitation of the current behavioral measures of WLT paradigms is that they do not control for interindividual differences in gastrointestinal capacity. Research has shown that a stomach with a large capacity requires a bigger meal to trigger satiation [22], wherefore it remains unclear if increased water ingestion during a WLT is attributable to a larger gastric capacity, to a less accurate perception of gastric changes, or both. Introduction of two separate drinking thresholds can, at least in part, circumvent this issue through the calculation of the percentage distribution of the volumes. Hence, we decided to include a second drinking step until subjectively perceived maximum stomach fullness. The relation of satiation to fullness allows to determine the percentage of maximum fullness at which satiation occurs. In other words, it represents a subjectively scaled measure of the interoceptive distance between the two thresholds, independent of absolute gastric capacity. Together with absolute water volumes, this complementary index provides a broader picture of gastric interoceptive sensitivity. To our knowledge, there is only one study that has directly compared two indices of sensitivity to gastric distention. Using a gastric balloon procedure, Geliebter and Hashim assessed maximum fullness alongside maximum capacity [21]. They found that, although bulimic patients needed larger volumes to produce maximum fullness and capacity, the ratio of the volumes did not differ between groups. The authors concluded that satiety as such may not be altered in bulimic patients, but that bulimics rather have larger gastric capacities. These results suggest that absolute volumes and their ratio provide differential and complementary information.

In light of this background, the primary purpose of the present study was to establish a standardized, two-step drink test to measure gastric interoception, consisting of two drinking periods assessing sensitivity to gastric satiation and maximum stomach fullness, and to provide normative data using a non-clinical adult sample. As previous research has been inconsistent with regard to the concepts measured, we included a self-report questionnaire assessing subjective sensations directly related to ingestion and gastric distention as behaviorally measured by the WLT paradigm. In accordance with studies showing that physiological responses arising from different visceral systems activate overlapping brain areas [1], and to further validate the WLT as a measure of gastric interoception, we investigated correlations between volumes of water ingested and cardiac interoceptive accuracy. In addition, relationships between WLT-II variables and eating disorder symptoms were explored.

## Materials and Methods

### Participants

Volunteer participants were recruited via advertisement from staff and students of the University of Luxembourg. To preclude gender effects on ingested water volumes during WLT [26,33] and heartbeat perception [34], we only included women in the current sample. Demographic data were collected for age, socioeconomic status, body weight and height, current and former illnesses, medication use, and physical activity. Exclusion criteria comprised intense physical activity, current or past mental disorders, and current physical conditions or medication that affect diet or weight. Inclusion and exclusion criteria were assessed using an in-house structured interview protocol that was developed based on validated instruments, such as the structured clinical interview for DSM-IV (SCID), the eating disorder examination (EDE), and the international physical activity questionnaire (IPAQ). The final sample consisted of 99 healthy female participants aged between 18 and 35 years (*M* = 22.86; *SD* = 3.41) with a mean BMI of 22.73 (*SD* = 3.60; range = 16.94–34.48). All participants provided written informed consent with all procedures being approved by the Ethics Review Panel of the University of Luxembourg.

### Cardiac interoceptive accuracy

Cardiac interoceptive accuracy was assessed using a heartbeat tracking task as described by Schandry [7]. A training interval of 25 seconds was followed by four experimental intervals of 25, 35, 45, and 55 seconds that occurred in a random order. During each interval, participants silently counted their heartbeats, while seated in a comfortable chair and instructed not to take their pulse or engage in any other manipulation that could facilitate heartbeat detection. Timing of counting phases and recording of participants’ reports of heartbeats was controlled by a stimulus presentation software. Electrocardiogram (ECG) was recorded throughout the whole procedure at a sampling rate of 1000 Hz through a BIOPAC^TM^ MP150 (Biopac Systems Inc., USA). The ECG raw signal was processed using the software Acqknowledge 4.2. Cardioceptive accuracy was determined across the four intervals using the following formula: Heartbeat detection score = 1/4 Σ [1 –(|recorded heartbeats–reported heartbeats|)/recorded heartbeats]. Heartbeat detection scores vary between 0 and 1, with higher scores representing a smaller difference between the numbers of reported and recorded heartbeats, i.e. higher cardioceptive accuracy. The heartbeat tracking task is a commonly used method to quantify interoceptive accuracy [10,35–38] that has shown good test-retest reliability [39] and has been found to correlate well with other heartbeat detection tasks [34].

### Water Load Test-II

The WLT-II was performed by asking participants to drink non-carbonated water at room temperature over two successive 5-min periods. During the first period, participants were instructed to drink water ad libitum until reaching the point of perceived satiation, that is, the sensation that determines meal termination. The following instruction was given in written form: ‘During the following five minutes, we ask you to drink water until perceiving a sign of satiation. By satiation we mean the comfortable sensation you perceive when you have eaten a meal and you have eaten enough, but not too much.’ During the second period, participants were asked to drink again, this time until reaching the point of maximum stomach fullness. The instruction read as follows: ‘We now ask you to drink again during five minutes. Please continue drinking until your stomach is completely full, that is, entirely filled with water.’ Participants were not told that there would be a second drinking phase in order not to influence their first water intake (i.e., to avoid that they would drink less in anticipation of the second drinking period). This 2-step drink test allows for calculating different WLT-II indices: (1) water volume (ml) required to produce satiation (sat_ml); (2) additional water volume needed to produce maximum fullness (Δfull_ml); (3) total water volume (total_ml), which is the sum of sat_ml and Δfull_ml; and (4) percentage of satiation to total volume (sat_%), which is calculated by dividing sat_ml by total_ml multiplied by 100, and represents an individual index of gastric interoception that is not confounded with stomach capacity. Hence, the WLT-II constitutes a multidimensional measure that describes different facets of gastric interoception.

Water was administered in non-transparent 5-liter flasks from which participants drank through a long straw to control for swallowing sizes. However, unbeknownst to the participants, the flask was filled with only 1.5 litres of water. This procedure blinded participants to the amount they consumed and gave them the impression of barely unlimited water supply while at the same time ensuring safety through the 1.5 litres maximum. After the first drinking period, the flask was substituted by a new but identically looking flask, again filled with 1.5 litres of water. The volume consumed from each flask in millilitres was recorded unobtrusively. The experimenter left the room during each drinking period to minimize experimenter effects.

### Questionnaires

#### WLT-II questionnaire

Items to assess subjective sensations related to the WLT were chosen based on previous studies using distension methods and on the eating disorder literature. Participants were asked to concentrate on their current abdominal sensations, especially if their stomach felt full or empty. They were asked to rate their momentary feelings of satiation and fullness, and completed eight questionnaire items measuring sensations of thirst, stomach tension, immobility, discomfort, guilt, sluggishness, nausea, and arousal. All items were answered on a 7-point scale ranging from 1 (no sensation/not at all) to 7 (extremely). Ratings were obtained before the first water intake (t0, baseline), and after the first (t1) and second (t2) drinking period.

#### Eating disorder inventory-2 (EDI-2)

The EDI-2 [40,41] assesses the specific psychopathology of eating disorders. It consists of 91 items, each of which is answered on a scale ranging from 1 (never) to 6 (always). In the present study, only the first three subscales were used: Drive for thinness (e.g., “I exaggerate or magnify the importance of weight”; seven items), measuring excessive concerns with dieting, preoccupation with weight, and fear of gaining weight; Bulimia (e.g., “I stuff myself with food”; seven items), assessing the tendency to consider and engage in episodes of uncontrollable overeating; Body dissatisfaction (e.g., “I think my thighs are too large”; nine items), referring to the degree of dissatisfaction with overall body appearance, as well as the size of different body parts. These three subscales were chosen because they assess core eating pathology typical of eating disorders, whereas the other subscales assess psychopathology commonly associated with, but not unique to, eating disorders [40]. The EDI-2 has been shown to have sound psychometric properties [40,42]: Drive for thinness had internal consistencies between .81 and .85 in different samples (α = .87 in our sample). Bulimia had internal consistencies between .83 and .87 (α = .79 in the present sample). For the subscale body dissatisfaction, internal consistencies of .91 have been reported (α = .88 in the current sample). Furthermore, Thiel and Paul [43] reported test-retest reliabilities between *r* = .81 and *r* = .89 for eating disordered participants.

#### Body consciousness questionnaire (BCQ)

The BCQ [44] measures the habitual tendency to focus attention on the body using three subscales: private body consciousness, public body consciousness and body competence. In the present study, only the private body consciousness (PBC) subscale was used. PBC emphasizes symptoms and measures the tendency to be attentive to internal bodily sensations (e.g., “I am sensitive to internal bodily tensions”; five items). Items are rated on a 5-point Likert scale from 0 (not at all characteristic) to 4 (extremely characteristic), with higher scores indicating a greater focus on internal bodily sensations. Moderately high internal consistencies were found for the PBC subscale in previous studies (α = .62) [45] and in the present sample (α = .62).

### Procedure

Participants were instructed to refrain from eating at least three hours before taking part in the experiment, and from drinking during the two hours prior to the session. All participants reported having complied with this instruction. Upon arrival, they were led into a sound-attenuated room, where a short interview was conducted to assess sociodemographic characteristics. They were then weighed and measured without shoes, before completing the heartbeat detection task. Participants were encouraged to use the rest room before the WLT-II was conducted. Subsequently, participants performed the WLT-II and completed the WLT-II questionnaires in the order described previously. Participants maintained a half-supine position throughout the experimental session. Finally, they completed the EDI-2 and the BCQ, together with other questionnaires not described here. A subset of participants (*n* = 28) repeated the WLT-II on a second occasion, one week after the initial testing session to investigate test-retest reliability.

### Data analysis

For normally distributed data (i.e., sat_%, heartbeat perception scores, EDI subscales drive for thinness and body dissatisfaction, private body consciousness), correlations were calculated using Pearson’s *r*, whereas correlations between non-normally distributed data (i.e., sat_ml, Δfull_ml, total_ml, BMI, EDI subscale bulimia) were determined using Spearman’s rho. To analyse the factorial structure of the WLT-II questionnaire, a principle component analysis (PCA) and varimax rotation was conducted. The number of factors was determined by Horn’s parallel analysis [46] in conjunction with visual inspection of the scree plot in order to prevent overfactoring [47]. In parallel analysis, the eigenvalues of empirical components are compared with those of components derived from random datasets with identical specifications (i.e., sample size, number of variables). Only factors with greater corresponding eigenvalues than those estimated from the random data were retained [48]. The effects of water intake on subjective sensations were analyzed using repeated-measures analyses of variance (ANOVA), followed, where appropriate, by Bonferroni-corrected paired-samples *t*-tests. Greenhouse-Geisser adjusted degrees of freedom were used in case of violation of the sphericity assumption.

## Results

### Normal values for ingested water volumes

Mean sat_ml was 428.36 ml (*SD* = 242.48; range: 134–1231 ml) and mean Δfull_ml was 306 ml (*SD* = 171.10; range: 58–868), adding up to a mean total_ml of 734.48 ml (*SD* = 316.80; range: 259–1683 ml). Furthermore, mean sat_% was *M* = 57.82 (*SD* = 16.33; range: 24.61–89.36).

### Ingested water volumes and heartbeat perception

Cardiac accuracy data from four participants was missing due to technical problems. The remaining 95 participants had a mean heartbeat detection score of .58 (*SD* = .19; range: .19–.98). This distribution of cardiac accuracy as measured by the heartbeat detection task is comparable to the distribution found in earlier studies in nonclinical samples [24,49].

Heartbeat detection scores correlated significantly negatively with sat_ml (*r* = -.30, *p* = .003), Δfull_ml (*r* = -.46, *p* < .001), and total_ml (*r* = -.46, *p* < .001). BMI was positively correlated with sat_ml (*r* = .30, *p* = .003), Δfull_ml (*r* = .22, *p* = .027), and total_ml (*r* = .36, *p* < .001). After adjustment for BMI, cardiac accuracy continued to be significantly correlated with sat_ml (*r* = -.26, *p* = .013), Δfull_ml (*r* = -.43, *p* < .001), and total_ml (*r* = -.45, *p* < .001). The heartbeat detection score was not related to sat_% (*r* = .17, *p* = .102) and sat_% was not correlated with BMI (*r* = .04, *p* = .710).

### Ingested water volumes and private body consciousness

PBC scores ranged from 0.60 to 4.00 (*M* = 2.35, *SD* = 0.74). There was no significant correlation between self-reported PBC and sat_ml (*r* = -.06, *p* = .564), Δfull_ml (*r* = -.07, *p* = .525) or sat_% (*r* = -.04, *p* = .706).

### Test retest of the water load test

Twenty-eight subjects underwent WLT-II on two occasions separated by one week. Correlations between sat_ml at the first session (WLT1) and at the second session (WLT2) were high (*r* = .62, *p* < .001). Participants drank significantly less water until feeling satiated at WLT2 compared to WLT1, *t*(27) = 2.82, *p* = .009, *d* = 0.53. Correlations between Δfull_ml at WLT1 and WLT2 were also large (*r* = .72, *p* < .001), and there was no significant difference between water volumes at WLT1 and WLT2, *t*(27) = 1.72, *p* = .096. Only medium to large test-retest correlations were found for sat_% (*r* = .47, *p* = .011), with no significant difference between sat_% at WLT1 and at WLT2, *t*(27) = .58, *p* = .566.

### Subjective ratings

For the WLT-II questionnaire, the Kaiser–Meyer–Olkin measure [50] revealed a value of .73 and Bartlett’s test of sphericity [51] was significant (*p* < .001), both suggesting that the dataset was adequate for factor analysis [52]. Parallel analysis and inspection of the scree plot indicated a one-factor solution. Only items with factor loadings ≥ .40 were considered [53], leading to the deletion of three items (thirst, stomach tension and immobility). The five remaining items (discomfort, guilt, sluggishness, nausea, and arousal) were subjected to another PCA, which confirmed the unidimensional structure. The final scale accounted for 51.75% of the variance and was labeled Negative Affect (NA). Its factor loadings ranged from .67 to .79. The corresponding Cronbach’s alpha values were .76 at baseline (t0), .77 after the first water intake (t1), and .72 after the second water intake (t2).

**Fig 1:**
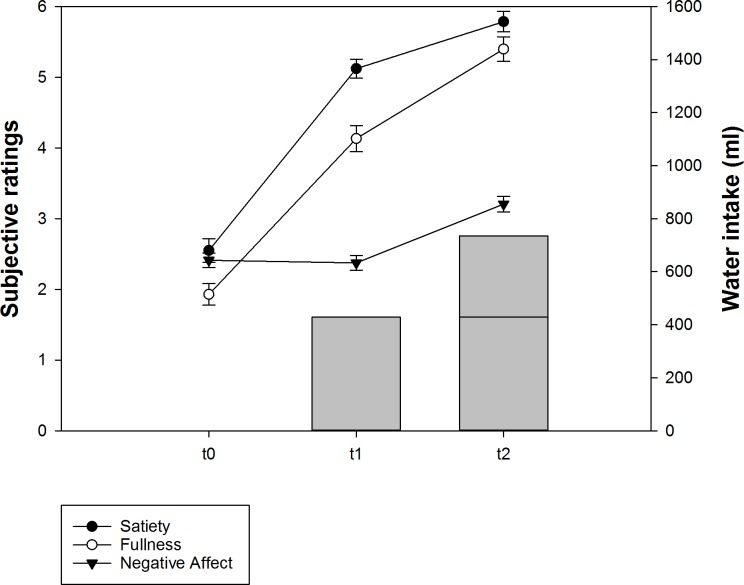
Mean (± SE) subjective sensations related to the WLT-II and ingested water volumes at t0, t1, and t2.

Mean values for satiety, fullness, and NA are depicted in Fig 1. Repeated measures ANOVA and post-hoc *t*-tests revealed increasing satiety and fullness ratings from t0 to t1 and from t1 to t2 (all *p*s < .001), thereby confirming sensitivity to the changes in ingested water volumes. Interestingly, NA was not affected by the first drinking period (*p* = .68), but a significant increase was observed after the second water intake (*p* < .001). These findings suggest that drinking until satiation was not experienced as aversive in the present sample and lend further credibility to the idea that ingestion until satiation and until fullness are conceptually different.

### Correlations with eating disorder pathology

Sat_ml was strongly positively related to the EDI subscale bulimia (*r* = .44, *p* < .001), and weakly positively correlated with drive for thinness (*r* = .22, *p* = .032), but not with body dissatisfaction (*r* = .16, *p* = .109). As for Δfull_ml, no significant correlations were found with bulimia (*r* = -.07, *p* = .503), drive for thinness (*r* = -.03, *p* = .792), or body dissatisfaction (*r* = .01, *p* = .894). Sat_% was positively related to bulimia (*r* = .38, *p* < .001), but not to drive for thinness (*r* = .17, *p* = .095) or body dissatisfaction (*r* = .12, *p* = .221).

Subjective ratings of fullness, satiety and NA were not related to the EDI subscales at t0 (bulimia: .02 < *r*s < .13, *p*s > .201; drive for thinness: .02 < *r*s < .18, *p*s > .082; body dissatisfaction: -.01 < *r*s < .09, *p*s > .378), t1 (bulimia: .00 < *r*s < .05, *p*s > .639; drive for thinness: -.04 < *r*s < .07, *p*s > .517; body dissatisfaction: -.09 < *r*s < .13, *p*s > .202) or t2 (bulimia: -.05 < *r*s < .12, *p*s > .259; drive for thinness: .00 < *r*s < .13, *p*s > .196; body dissatisfaction: -.05 < *r*s < .11, *p*s > .268).

## Discussion

Impaired perception of interoceptive cues is a frequently suggested abnormality in eating disordered patients. However, the currently available tests to quantify interoception in the eating domain are nonspecific, unstandardized, and yield heterogeneous results. In the present study, we aimed at developing a standardized test to measure gastric interoception and to evaluate it in a non-clinical sample. Importantly, the WLT-II was designed to be easy to apply, well tolerated, and should improve upon some of the limitations noted for the initial version.

Our first main observation, in some ways a test of construct validity, was that both the ingested volume of water required to produce satiation and maximum fullness were significantly negatively related to cardiac accuracy. In addition, both ingested volumes were related to BMI. A positive association between BMI and maximal ingested volume in healthy controls has also been reported in previous research [33,54]. Our findings reinforce and extent results from previous studies, which indicate that sensitivity for interoceptive processes may be generalized, at least across cardiovascular and gastric domains [24,55]. Similar to the present findings, Herbert and colleagues reported that individuals with high heartbeat detection scores consumed significantly less water until feeling first signs of fullness [24]. In their study, however, feelings of fullness were not directly associated to an eating-related context and only one drinking threshold was used, thereby not differentiating levels of satiation and stomach fullness. Our results extend these findings by indicating that cardiac interoceptive signal perception is rather associated with sensations of fullness than with eating-related satiation. This observation has implications for future studies on gastric interoceptive ability. An additional explanation is that water quickly empties from the stomach, which could have contributed to the seemingly weaker association between the first drinking volume and cardiac sensitivity. Indeed, gastric emptying of a liquid starts within minutes of water ingestion [56], so that part of the water ingested at the beginning of the WLT-II (i.e., during the first drinking period) may have emptied immediately to the duodenum, which could have influenced gut perception and thereby intake until satiation [57].

In the present study, percentage of satiation to maximum fullness was not related to heartbeat detection scores. This is little surprising, because the two measures are conceptually different; while heartbeat detection tasks assess individual differences in cardiac interoceptive accuracy, sat_% adopts a within-individual perspective on how ‘close’ satiation is to fullness (in percent) for a given individual, irrespective of his/her actual gastric capacity (in ml). An additional explanation for the weak (i.e., sat_ml) or inexistent (i.e., sat_%) correlations between WLT-II indices and cardiac accuracy is provided by the WLT-II’s focus on interoceptive ability in an explicitly eating-related context that is directly linked to “somatic markers” associated with food intake. Validity of the WLT-II is also supported by self-reported subjective sensations in response to drinking periods. Satiety and fullness ratings increased as a consequence of water consumption, which further substantiates the suitability of the WLT-II to gradually induce satiation and gastric fullness. Similarly, previous studies have demonstrated that gastric distention in the absence of caloric intake (i.e., by inserting gastric balloons and filling them with water) increases sensations of fullness [21,58]. It has, however, been questioned whether these findings were due to the discomfort associated with the placement and inflation of the balloons, rather than normal feelings of fullness. The present findings add to this line of research by showing that sensations of satiation and fullness may be influenced by non-invasively loading the stomach with water. The results from the factor analysis performed on the WLT-II questionnaire led to a unidimensional structure of the scale, which was named “negative affect”. It has to be noted, however, that these results are based on a small sample size, and that additional studies should be conducted to replicate the psychometric properties of the measure. Nevertheless, our results revealed that NA was not affected by drinking until satiated, yet a significant increase in NA was observed after the second drinking period. This is not surprising, as the NA subscale is mainly composed by items referring to states of discomfort, including both psychological and physiological factors. This result further confirms the distinction between satiation reflecting a positive, comfortable sensation, in contrast to maximum fullness, which has clearly been found to cause negative subjective sensations. In addition, no participant refused to drink during the second period, which further confirms that satiation and maximal fullness are well discriminable interoceptive states.

The amount of water ingested was uncorrelated with the PBC subscale measuring the general tendency of an individual to be attentive to internal bodily sensations. This finding is in line with previous research on cardiac accuracy that failed to find an association between self-reported awareness and experimentally measured visceral sensitivity [59,60]. Accordingly, Garfinkel and colleagues proposed a dimensional construct of interoception that distinguishes between different levels of interoception. In particular, they introduced the distinction between interoceptive accuracy (i.e., the accurate detection of bodily sensations, as measured by objective tests of interoceptive proficiency) and interoceptive sensibility (i.e., the overall tendency to focus on internal bodily sensations, measured using self-report questionnaires), which are two components that seem to represent distinct processes that should not be conflated [38,61].

Although results were based on a small sample size and should be considered preliminary, they indicated satisfactory repeatability of the WLT-II over time for both drinking periods. Test-retest correlations for the WLT-II were slightly lower than those reported for the heartbeat detection task [62] and comparable to the values achieved using a standard 5-minutes WLT [26]. Stability of the WLT-II was, however, challenged by the differences in water volumes consumed between the first and the second session. Indeed, participants drank significantly less water until feeling satiated at the second compared to the first session. This is most probably due to the fact that, during the first session, participants were unaware that they would have to drink twice, whereas at re-test they remembered the procedure. Hence, knowing that there would be a second drinking phase, some individuals might have consumed less water until satiation at re-test. Lower water volumes at re-test were also reported by Jones and colleagues using a traditional 5-minutes WLT [26]. These results suggest that the WLT-II is prone to the within-subject variation observed with other measures of gastric function, especially using liquid stimuli [63,64]. Future studies should investigate if informing participants from the beginning about the second drinking phase leads to lower within-subject variation. This procedure could possibly be more appropriate when monitoring changes in gastric interoception over time, for example when assessing therapeutic progress in eating disordered patients. Furthermore, high variability in WLT-II variables across time could also be an indicator for poor gastric interoception. Future research should explore this possibility in more detail.

Across this non-clinical sample of young adults, our data shows positive correlations between WLT-II variables and eating disorder psychopathology. Most notably, satiation volume was strongly positively related to bulimic symptoms, whereas no noteworthy correlations were found for fullness volume. These findings suggest that individuals with bulimic symptoms (i.e., binge-eating and purging behaviors) either need considerably larger volumes until the onset of satiation, or are less sensitive to gastric signals and therefore drink beyond the satiation threshold. This question that especially shows up when comparisons in gastric interoception between different groups of eating disordered, obese, and healthy individuals are intended, may be addressed by examining the percentage distribution of the volumes. Percentage of satiation to total volume was positively related to bulimic symptoms, indicating that in individuals with high bulimia scores, satiation is reached at a larger proportion of maximum stomach fullness. These results suggest that satiation may be disordered in individuals high in bulimic symptoms, as they stopped drinking at a point that was closer to their (subjective) gastric capacity. In contrast, Geliebter and Hashim found no difference in the ratio of the volumes between binge-eating patients and control subjects [21]. In their study, however, they assessed maximum fullness and maximum discomfort volumes, in contrast to more eating-related satiation and maximum stomach fullness in the present study. Also, they induced gastric distention using an invasive, gastric balloon method, which could account for the differing findings.

Taken together, the present results suggest that, depending on their instructions, WLT-II indices are associated with either the commonly utilized heartbeat detection task to measure objective interoceptive sensitivity, or with eating disorder-related symptoms. While the newly introduced, eating-related indices sat_ml and sat_% correlate with bulimic symptoms, maximum fullness was rather related to cardiac accuracy, thereby replicating previous findings by Herbert and colleagues [24]. Future studies should focus on further investigating the correlates of these different facets of gastric interoception. As outlined in the introduction, previous research investigating interoceptive processing in the eating domain mostly relied on emotional and cardiac perception and yielded contradictory findings. Those studies directly measuring gastric perception have used differing methods. For example, development of satiation and meal termination have frequently been examined using different laboratory test meals [3,4]. Caloric meals introduce a variety of factors that are difficult to control for, such as caloric composition, osmolality, palatability, and consistency [26]. Water, on the other hand, offers a modality that restricts satiation determinants to gastrointestinal distention, visceral sensations, and psychological concomitants of fullness. Especially during and shortly after food intake, when overeating may occur, sensations of satiation are primarily determined by gastric distention rather than by the nutrient content of a meal [65]. Gastric distension triggers mechanosensitive receptors that in turn relay their information via vagal afferents [66] to the central nervous system [67], thereby regulating satiation and food intake. Accordingly, it has been demonstrated that meal volume, but not energy content, affected perceptions of fullness and satiety in healthy participants [68]. These observations, together with the present findings, suggest that loading the stomach with water represents a standardized and non-invasive method to investigate the development and perception of satiation and stomach fullness.

We do not claim that the WLT-II is an accurate measure of gastric volume, because there is a number of confounding variables that this test cannot account for. Hence, the WLT-II was not designed to assess stomach capacity, but it was rather developed as a standardized and non-invasive test to measure sensitivity for gastric functions, irrespective of stomach volume. With its multiple indices, consisting of behavioral and subjective measures, the WLT-II provides a comprehensive and multifaceted measure of gastric interoception. Although the validity and correlates of these different indices need further investigation, the present results suggest that the WLT-II is an easily performed, well-tolerated and reliable test, comprising variables that correlate with both performance accuracy on the heartbeat tracking task and eating disorder-related symptoms. Importantly, the WLT-II could emerge as a useful clinical tool to measure interoceptive processing in eating disorders and obesity.

## Supporting Information

S1 Dataset Raw data used in the present article. (SAV)
